# An Evaluation of Moderate-Refractive-Index Nanoantennas for Enhancing the Photoluminescence Signal of Quantum Dots

**DOI:** 10.3390/nano14221822

**Published:** 2024-11-14

**Authors:** Rafael Ramos Uña, Braulio García Cámara, Ángela I. Barreda

**Affiliations:** Department of Electronic Engineering, University Carlos III of Madrid, Avda. de la Universidad, 30, 28911 Leganés, Spain; brgarcia@ing.uc3m.es

**Keywords:** plasmonic, quantum dots, moderate refractive index, high refractive index, single-photon sources

## Abstract

The use of nanostructures to enhance the emission of single-photon sources has attracted some attention in the last decade due to the development of quantum technologies. In particular, the use of metallic and high-refractive-index dielectric materials has been proposed. However, the utility of moderate-refractive-index dielectric nanostructures to achieve more efficient single-photon sources remains unexplored. Here, a systematic comparison of various metallic, high-refractive-index and moderate-refractive-index dielectric nanostructures was performed to optimize the excitation and emission of a CdSe/ZnS single quantum dot in the visible spectral region. Several geometries were evaluated in terms of electric field enhancement and Purcell factor, considering the combination of metallic, high-refractive-index and moderate-refractive-index dielectric materials conforming to homogeneous and hybrid nanoparticle dimers. Our results demonstrate that moderate-refractive-index dielectric nanoparticles can enhance the photoluminescence signal of quantum emitters due to their broader electric and magnetic dipolar resonances compared to high-refractive-index dielectric nanoparticles. However, hybrid combinations of metallic and high-refractive-index dielectric nanostructures offer the largest intensity enhancement and Purcell factors at the excitation and emission wavelengths of the quantum emitter, respectively. The results of this work may find applications in the development of single-photon sources.

## 1. Introduction

Single-photon sources are a crucial components in the development of photonic integrated circuits for quantum information processing and communication. These sources must generate individual photons on demand with high efficiency, purity, and indistinguishability to enable scalable quantum technologies [1,2,3]. Among other alternatives, semiconductor quantum dots (QDs) are promising candidates for such single-photon sources due to their optical properties, such as narrow emission linewidths, high photon extraction efficiencies, and the potential for electrical injection [4,5,6]. Additionally, QDs can be engineered to emit at desired wavelengths by tuning their size and composition, making them suitable for various applications in quantum optics and photonics [7,8]. In particular, QDs emitting in the visible range can be fabricated using various semiconductor materials, such as CdSe, CdTe, InP [9] or, recently, perovskites [10]. These QDs can be synthesized using colloidal chemistry techniques, which allow for precise control over their size, shape and composition, and they can even be embedded in a wide range of host materials, such as polymers or silica, to improve their stability and ease of integration into photonic devices [11,12]. Despite the promising properties of visible-range QDs for single-photon sources, the stringent requirements for their practical applications still require remarkable efforts to improve their efficiency and purity [13]. In this sense, one of the main challenges is to enhance the spontaneous emission rate of QDs, which determines the photon generation efficiency and timing jitter. This can be achieved by exploiting the Purcell effect, which occurs when the QD is coupled to a resonant nanostructure that modifies the local density of optical states (LDOS) experienced by the emitter. In this regard, there are a huge number of proposals in the state of the art considering different materials and geometries [14,15,16,17,18]. In particular, plasmonic nanostructures are well known for their capacity to confine light into deep subwavelength volumes, leading to strong field enhancements and large Purcell factors. This enhancement can increase the spontaneous emission rate of QDs, improving their photon generation efficiency and reducing their time response [19,20]. However, plasmonic structures also introduce additional losses, which can degrade the quantum efficiency and indistinguishability of the emitted photons. An alternative approach is to use high-refractive-index (HRI) dielectric nanostructures, which can support Mie resonances and exhibit low ohmic losses in the visible range compared to plasmonic structures [21,22]. These resonances can also lead to strong field enhancements and large Purcell factors, while maintaining high quantum efficiencies. Furthermore, the resonant frequencies of dielectric nanostructures can be tuned by varying their size, shape and material composition, allowing for optimal coupling with QD emission. Additionally, dielectric nanostructures can be fabricated using well-established semiconductor processing techniques, making them compatible with existing photonic integrated circuit technologies [23,24]. However, the field enhancement in dielectric nanostructures is typically lower than in plasmonic structures due to the weaker light confinement. To overcome this limitation, researchers have explored the use of hybrid plasmonic–dielectric nanostructures, which combine the advantages of both materials [25,26,27,28]. These structures can exhibit remarkable field enhancements while maintaining low losses, making them promising candidates for enhancing the performance of QD-based single-photon sources. Recently, a new alternative has arisen with the use moderate-refractive-index (MRI) dielectric nanostructures [29]. MRI (1.7<n<3) dielectric nanoparticles (NPs), typically made from materials like silicon nitride $\left({\mathrm{Si}}_{3}N_{4}\right)$ or titanium dioxide $\left({\mathrm{TiO}}_{2}\right)$, offer several advantages over their HRI counterparts, such as being chemically synthesized and flexibly assembled [30]. The resonances of MRI are located at shorter wavelengths in comparison with those of HRI dielectric nanospheres with the same size and shape, because their spectral evolution, mainly their magnetic dipolar resonance, follows the formula λ=nd, with λ being the resonant wavelength, *n* the refractive index, and *d* the diameter of the nanosphere [31]. Moreover, previous studies have already demonstrated that MRI dielectric NPs show broad Mie resonances in the visible spectral range. In [32], it was evidenced that nanospheres with a refractive index of 2.2, i.e., synthesized hydrogenated amorphous silicon (a-Si:H), show broad resonant conditions due to the overlapping of the dipolar electric and magnetic Mie modes, leading to the emission enhancement of several excitons of 2D materials located at the bottom of the MRI NP. Here, we propose a comparative study of different nanostructures to optimize both the excitation and emission of single QDs. In particular, the proposed structures consist of homogeneous and hybrid dimers made of metallic, MRI and HRI dielectric materials. The results of this work can help in the future design, fabrication and development of more efficient single-photon sources.

## 2. Methodology

**Figure 1:**
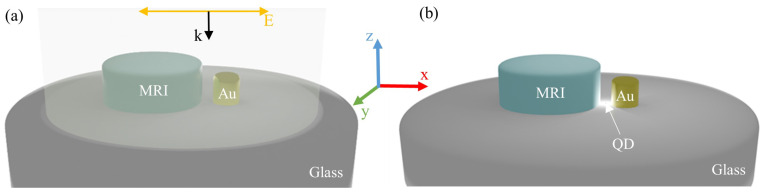
(**a**) A scheme of the illumination of the dimer by a plane wave propagating along the negative direction of the *z*-axis, i.e., from the air to the substrate, and linearly polarized parallel to the dimer orientation (*x*-axis). (**b**) A scheme of the dimer when it is excited by an electric dipole oscillating in the *x*-direction, representing the QD, located in the middle of the gap and 5 nm above the substrate to take into account the QD size.

The presented results were obtained by means of the commercial software COMSOL Multiphysics V6.2, which employs the Finite Element Method (FEM) to solve Maxwell equations by establishing the boundary conditions of the system. The considered geometry for the nanoantenna consists of a dimer of nanocylinders. As can be observed in Figure 1, two different cases were analyzed. On the one hand, homogeneous dimers of metallic, MRI or HRI dielectric NPs. On the other hand, hybrid nanostructures combining metallic and dielectric NPs. Silicon and gold were used as the HRI and metallic materials, respectively, with their theoretical optical properties obtained from Palik [33], while MRI had a constant refractive index of 2.2 in the whole analyzed spectral range, like that described in [32], which corresponds to synthesized hydrogenated amorphous silicon. Glass was used as the material for the substrate, with a refractive index of 1.5. The dimer configuration was set to enhance the excitation and emission of a quantum emitter, corresponding to a QD located in the middle of the gap between the NPs and on the substrate, as this may be the most suitable location for the QD, from an experimental point of view. The relative position of the QD with respect to the nanocylinders over the substrate was also tested and analyzed, and the results are shown in the Supplementary Information (SI-3). Other QD positions that were not lying on the substrate were not considered because they were experimentally challenging. The gap was set to 20 nm wide in order to simulate a realistic practical situation, as it is feasible for QDs to be fabricated by electron beam lithography (EBL). The QD was established to have a radius of 5 nm according to the sizes provided in the literature [34,35,36,37]. Specifically, a CdSe/ZnS QD was considered, whose excitation and emission wavelengths were centered at 570 and 650 nm, respectively [37,38].

The most suitable radii and heights for the NPs of the dimers were selected with the main objective of obtaining plasmonic or Mie resonances at the excitation and emission wavelengths of the QD. To have an idea of the approximate sizes of the NPs in the dimer, we firstly determined the sizes of the single metallic and dielectric NPs showing dipolar resonances in the spectral region of interest. In particular, the multipolar decomposition for every selected NP was performed in order to atain the mode contributions to the scattering efficiency. The different contributions were retrieved by integrating the displacement currents induced within the NPs [39]. The details for the multipolar decomposition from the simulations are described in the Supplementary Information SI-1. It is important to mention that the multipolar decompositions were performed in a homogeneous medium, without the substrate. Once we had selected the sizes that were best adapted for every material, the dimers were studied in terms of excitation and emission enhancement considering a glass substrate. The presence of the substrate led to a small red-shift of the resonances with respect to previous results, which was taken into account. However, as these structures present broadband Mie resonances, this effect did not significantly impact the results. A discussion of this, along with some possible fabrication challenges, is provided in Supplementary Information SI-4.1 and SI-4. During this research, a large range of sizes was studied, but due to the lack of space, only the most remarkable ones were considered. For further information about size dependence, a comprehensive study is provided in Supplementary Information SI-2.

**Figure 2:**
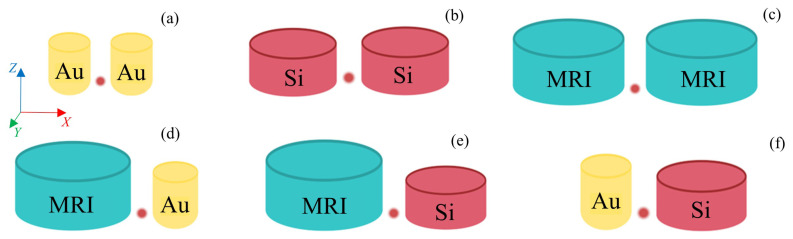
Scheme of all different dimer configurations. In (**a**–**c**), non-hybrid (homogeneous) dimers are represented: (**a**) two gold NPs, (**b**) two silicon NPs and (**c**) two MRI NPs. And in (**d**–**f**), hybrid dimers combining all three previous materials are shown. The red dot between the NPs represents the CdSe/ZnS QD.

The excitation enhancement is given by the square module of the electric field enhancement ${\left|E\right|}^{2}/{\left|E_{0}\right|}^{2}$ at the excitation wavelength; here, |E| is the electric field amplitude averaged in the volume of the QD, and $|E_{0}|$ corresponds to the electric field amplitude of the incident plane wave averaged in the same volume. On the other hand, the emission enhancement is provided by the Purcell factor (*F*). As both excitation and emission enhancements were evaluated, two sets of simulations were carried out to analyze the performance of the different proposed geometries (see Figure 2). For the calculation of the scattering efficiency spectra and the electric field enhancement at the QD position due to the nanoantenna, the NPs were illuminated by a plane wave linearly polarized along the *x*-axis, i.e., the axis that joins both NPs of the dimer configuration, and propagating along the negative direction of the *z*-axis, i.e., the disk axis. Meanwhile, to assess the Purcell factor and radiation efficiency, it was required to incorporate the QD into the simulations. The QD is represented by an electric point dipole source, with a dipole moment oriented along the *x*-axis, placed in the gap between the NPs and 5 nm above the substrate to take into account the QD size, as described before. According to the Purcell effect, the spontaneous emission rate of a quantum emitter can be modified due to its interaction with a resonant optical cavity [40]. In particular, the total decay rate of a quantum emitter inside a cavity is driven by the Purcell factor $F=3/{\left(4\pi \right)}^{2}Q/V$, where *V* is the volume normalized by the wavelength λ over the local mode refractive index *n* cubed ${\left(\lambda /n\right)}^{3}$, and *Q* is the resonator quality factor [40,41,42,43,44]. To assess the Purcell factor, which is related to LDOS, the following expression was considered:

(1) $$F=\frac{P_{r}+P_{\mathrm{nr}}}{P_{r}^{0}+P_{\mathrm{nr}}^{0}}$$

where $P_{r}$ is the radiative power, $P_{\mathrm{nr}}$ the non-radiative power, and $P_{r}^{0}$ and $P_{\mathrm{nr}}^{0}$ their intrinsic counterparts; this is when the nanostructures are not considered. This relation describes the total power emitted by the dipole along with the nanoantenna over the power emitted by the dipole in air. The radiation efficiency is calculated as the radiative power over the total emitted power (radiative and non-radiative):

(2) $$\eta =\frac{P_{r}}{P_{r}+P_{\mathrm{nr}}}$$

To perform the COMSOL simulations, a sphere of radius 800 nm surrounded the geometry, in order to calculate the scattering cross-section and the radiative power by integrating the Poynting vector at its surface. The non-radiative power was obtained by integrating the ohmic losses in the volume of the NPs. A sphere of radius 1200 nm was divided into two halves; the upper one was made of air, and the lower one was made of the substrate refractive index. This sphere was surrounded by a perfectly matched layer (PML) of thickness λ/4, being λ the wavelength of the incident radiation. As previously mentioned, for the scattering efficiency and electric field enhancement simulations, a plane wave was chosen as the illumination source. However, for the Purcell factor calculations, a dipole source was used. To get the convergency of the results, when the dipolar source was included, a sphere of radius 5 nm surrounding the dipole was created. Its mesh size corresponded to 0.6 nm at maximum element size and 0.03 nm at minimum.

## 3. Results and Discussion

A comparative study between metallic, HRI and MRI dielectric materials for a nanocylindrical dimer was performed in order to increase the Purcell factor, radiation efficiency and electric field enhancement. Larger enhancements are achieved when the gap between the NPs is reduced; however, the gap considered was 20 nm due to experimental constraints. Also, the sizes of the NPs were limited in the range of 20–200 nm in height and 50–300 nm in radius, due not only to manufacturing limitations, but also to the necessity of staying in a subwavelength range.

### 3.1. Single Nanoparticles

**Figure 3:**
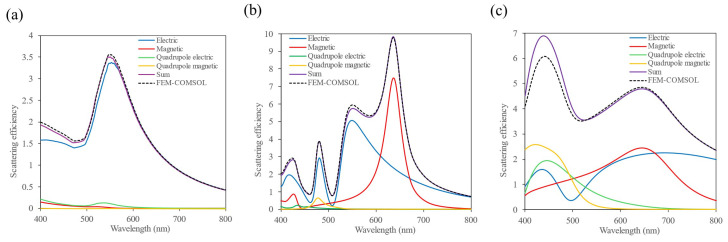
The scattering efficiency and multipolar decomposition for metallic and dielectric isolated NPs in air. The nanoparticles were illuminated by a plane wave propagating along the negative direction of the *z*-axis (parallel to the disk axis) and linearly polarized along the *x*-axis (perpendicular to the disk axis). (**a**) A gold NP of *R* = 50 nm and *H* = 150 nm, (**b**) a silicon NP of *R* = 120 nm and *H* = 80 nm and (**c**) a MRI NP of *R* = 150 nm and *H* = 200 nm. Black dotted line: the scattering efficiency obtained with COMSOL. Purple solid line: the scattering efficiency obtained as the sum of the dipolar electric and magnetic, and quadrupolar electric and magnetic, contributions. Blue and red solid lines: dipolar electric and magnetic contributions, respectively. Green and yellow solid lines: quadrupolar electric and magnetic contributions, respectively.

Several sizes for the single metallic, HRI and MRI NPs were considered in order to attain the appropriate radius and height of the NP that provides dipolar resonances at the excitation and emission wavelengths of the QD. However, for simplicity, only the results for the chosen dimensions are represented. In Figure 3 the multipolar decomposition is shown for a metallic NP of radius *R* = 50 nm and height *H* = 150 nm, a HRI dielectric NP of *R* = 80 nm and *H* = 120 nm, and a MRI dielectric NP of *R* = 150 nm and *H* = 200 nm. As observed in Figure 3, while the multipolar decomposition for the gold NP only shows an electric response, for the dielectric ones, both magnetic and electric dipolar resonances are attained. Focusing on the regions of interest, i.e., in the regions of 570 nm and 650 nm, the dipolar response dominates. For the gold NP, an electric dipolar resonance is observed at the excitation wavelength. In the case of the HRI dielectric NP, at the excitation and emission wavelengths, the main contributions to the scattering efficiency come from the dipolar electric and magnetic resonances. Conversely, for the MRI dielectric NP, the dipolar electric and magnetic resonances are broader than for the HRI dielectric one, achieving almost the same contribution of both resonances to the total scattering in the spectral range between 550 and 700 nm.

### 3.2. Homogeneous Dimers

The sizes of the single NPs were optimized to achieve a dipolar electric and magnetic response within the wavelength range of interest, as explained. Therefore, the dimensions were kept constant when forming the dimers. Despite the fact that both the substrate’s presence and the NP interaction result in a red-shift in the resonances, the scattering of the dimers adjusts to the desired wavelength range.

#### 3.2.1. Electric Field Enhancement and Scattering Efficiency Spectra

**Figure 4:**
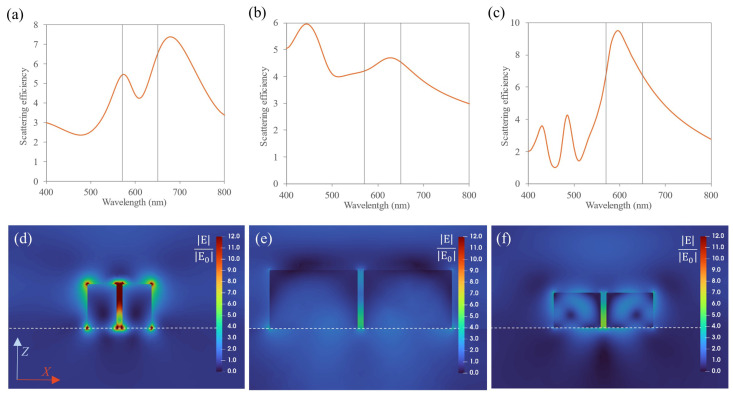
(**a**–**c**) Scattering efficiency for homogeneous dimers: (**a**) Au-Au, (**b**) MRI-MRI and (**c**) Si-Si dimers. The gray vertical lines correspond to the excitation and emission wavelengths. (**d**–**f**) Near-field maps (Z-X plane) at the excitation wavelength (570 nm) for the homogeneous dimers: (**d**) Au-Au, (**e**) MRI-MRI and (**f**) Si-Si. A hot-spot is observed in between the NPs, specified as the reddish/greenish area. The dashed white line represents the position of the substrate.

In line with the previous section, the scattering efficiency spectra were evaluated for the homogeneous dimers. In Figure 4a–c, it is observed that the scattering efficiency spectra for the dimers are broader than for the isolated NPs due to the interaction effects between both components of the dimer and the substrate’s presence. Furthermore, resonances were attained in between the excitation and emission wavelengths, leading to high scattering efficiencies at λ = 570 nm and λ = 650 nm. Comparing Figure 4b,c, it is evidenced that the MRI dimer show broader resonances but lower scattering efficiencies. Focusing on the electric field enhancement (see Figure 4d–f, where the near-field maps are represented in the scattering plane (Z-X) at the excitation wavelengths for the corresponding dimers), hot-spots were observed in the gap between the NPs. The highest hot-spot is obtained for the gold dimer. For the dielectric nanostructures, although most of the radiation is confined inside the nanostructure for single NPs, when they conform dimers, hot-spots can also be achieved in the gap, being this the main reason for analyzing dimers instead of single NPs. Specifically, from the near-field maps, it is shown that a hot-spot is localized in between the NPs encompassing the volume of the QD. The results for the average field enhancement over the volume of the QD are shown in Table 1.

#### 3.2.2. Purcell Factor and Radiation Efficiency

Concerning the study of emission enhancement of the QD, an electric dipole located in the gap between the NPs of the dimer was used as the light source, as described in the methodology section. The presence of an antenna usually boosts the LDOS at the emission wavelength, decreasing the radiative lifetime. In particular, a high Purcell factor is desired to accelerate the emission process. Moreover, large radiation efficiency is also requested in order to attain efficient single quantum emitters. In Table 1, the Purcell factor and the radiation efficiency for the different studied dimers are summarized. The Purcell factor, calculated as described in Equation (1), demonstrates that the emission of the QD is more significantly influenced by the presence of the gold dimer than by the dielectric NPs. Specifically, the Purcell factor is almost 9 times larger for gold than for silicon, and more than 37 times larger than for the MRI dimer. In contrast, regarding the radiation efficiency, the highest values were obtained for the dielectric materials, due to the low losses in comparison with the metallic ones that suffer the Joule’s effect. It is worth noting that the MRI NPs are considered to have a negligible imaginary part of the refractive index (*k* = 0), which is the reason for the 100% radiative emission. The product of the excitation (square of the electric field enhancement) and emission enhancement (Purcell factor) is shown in the last column of Table 1. Based on these results, it is concluded that the performance of homogeneous MRI nanodimers is not as good as that of metallic or HRI nanoantennas for enhancing the efficiency of quantum emitters.

### 3.3. Hybrid Dimers

Hybrid nanostructures were analyzed due to their ability to combine the best properties from dielectric and metallic NPs, which include strong confinements of electromagnetic energy in the surroundings of metallic NPs and low losses of dielectric ones.

#### 3.3.1. Electric Field Enhancement and Scattering Efficiency Spectra

**Figure 5:**
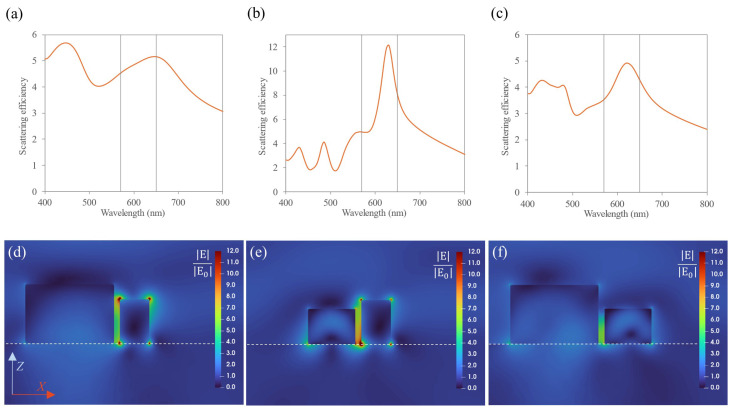
(**a**–**c**) Scattering efficiency for hybrid dimers: (**a**) MRI-Au, (**b**) Si-Au and (**c**) MRI-Si dimers. The gray vertical lines correspond to the excitation and emission wavelengths. (**d**–**f**) Near-field maps (Z-X plane) at the excitation wavelength (570 nm) for the hybrid dimers: (**d**) MRI-Au, (**e**) Si-Au and (**f**) MRI-Si. The dashed white line represents the position of the substrate.

The scattering efficiency for each material combination is represented in Figure 5a–c, where, again, the combinations involving a MRI dielectric NP show broad Mie resonances at the desired wavelength range, but higher scattering efficiencies are reached when employing the HRI dielectric NPs. Near-field maps at an excitation wavelength (570 nm) are plotted in Figure 4d–f. The highest hot-spots are observed in the corners of the NPs due to the lightning rod effect. However, hot-spots are still achievable at the QD position. In Table 2, the results for the average field enhancement over the volume of the QD are shown, which demonstrate the largest enhancement attained for the gold–silicon combination.

#### 3.3.2. Purcell Factor and Radiation Efficiency

The results regarding the Purcell factor and radiation efficiency for these hybrid combinations are shown in Table 2. Additionally, a comparison between these results and previous studies is included in the Supplementary Information (SI-5). It is firstly observed that the integration of MRI with either silicon or gold increases the product of the excitation and emission enhancement with respect to the MRI dimer, but it is still lower than the HRI–gold dimer combination. Therefore, while MRI NPs can be used to enhance QDs, they are not the most optimal choice compared to metals and HRI dielectrics such as gold and silicon.

## 4. Conclusions

Studies have demonstrated that metallic and high-refractive-index dielectric nanoparticles are effective in enhancing the emission of quantum emitters, with important applications in quantum communications and quantum information. However, the case of moderate-refractive-index dielectric materials remains unexplored with regard to this objective. In this work, we carried out a systematic comparison of homogeneous and hybrid dimers of metallic, high-refractive-index and moderate-refractive-index dielectric nanoparticles for enhancing the excitation and emission of a quantum dot located in the gap between the nanoparticles. Firstly, we optimized the size of the single metallic, high-refractive-index and moderate-refractive-index dielectric nanoparticles to attain dipolar resonances in the wavelength range of interest. Secondly, homogeneous and hybrid dimers were analyzed for a gap no smaller than 20 nm due to fabrication limits. It was observed that although overlapping of electric and magnetic Mie resonances appears for the moderate-refractive-index dielectric materials, leading to broader resonances than those attained for metallic or high-refractive-index dielectric nanoparticles, the excitation and emission enhancement is lower than for those other materials. In particular, the largest electric field enhancements at the position of the quantum dot and Purcell factors are attained for the gold dimer. However, low radiation efficiencies are reported due to ohmic losses. Silicon dimers provide lower excitation enhancement and Purcell factors than the gold dimers; nevertheless, this low value is compensated for by the larger radiation efficiency due to the low losses. Regarding the hybrid nanostructures, the combination of silicon–gold provides better results than those involving moderate-refractive-index dielectric nanoparticles. This leads us to conclude that moderate-refractive-index dielectric nanoparticles do not perform as well as metallic and high-refractive-index dielectric materials in increasing the efficiency of single quantum emitters. This work may serve as a guide for obtaining more efficient single quantum emitters, depending on their application. On the one hand, for studies where the emission enhancement of several excitons is required, moderate-refractive-index dielectric nanoparticles can be valuable structures due to their broadband electromagnetic response. On the other hand, in studies focusing on the enhancement of quantum dots, specific wavelengths for excitation and emission are sought; therefore, high-refractive-index dielectric nanoparticles can provide higher values as long as the excitation and emission wavelengths overlap with the resonances of the nanoparticles.

## Figures and Tables

**Table 1:** Purcell factor (*F*), radiation efficiency (η), averaged electric field enhancement $\left|E|/\right|E_{0}|$ at the position of the QD and product of the excitation and emission enhancement $\left(|E|/\right|E_{0}{\left|\right)}^{2}$·*F* for the considered homogeneous dimers. For a more direct comparison, the NP sizes used in all geometries are the same as those selected for every material in Figure 3.

	Materials	NP1		NP2		F	η	$\left|E|/\right|E_{0}|$	$\left(|E|/\right|E_{0}{\left|\right)}^{2}\cdot F$
		$R_{1}$ [nm]	$H_{1}$ [nm]	$R_{2}$ [nm]	$H_{2}$ [nm]				
1	Au-Au	50	150	50	150	285.64	0.66	8.612	21,185
2	MRI-MRI	150	200	150	200	7.63	1	3.18	77
3	Si-Si	80	120	80	120	31.80	0.96	5.39	922

**Table 2:** The Purcell factor (*F*), radiation efficiency (η), averaged electric field enhancement $\left|E|/\right|E_{0}|$ at the position of the QD and product of the excitation and emission enhancement $\left(|E|/\right|E_{0}{\left|\right)}^{2}$·*F* for the considered hybrid dimers. The sizes of the NPs remain constant as in Figure 3 and Table 1 for a more direct comparison.

	Materials	NP1		NP2		F	η	$\left|E|/\right|E_{0}|$	$\left(|E|/\right|E_{0}{\left|\right)}^{2}\cdot F$
		$R_{1}$ [nm]	$H_{1}$ [nm]	$R_{2}$ [nm]	$H_{2}$ [nm]				
4	MRI-Au	150	200	50	150	109.47	0.66	4.64	2361
5	MRI-Si	150	200	80	120	18.31	0.97	4.06	302
6	Si-Au	80	120	50	150	138.39	0.72	7.44	7660

## Data Availability

Under reasonable request, the author will provide the datasets created and/or analyzed during the current investigation.

## Supplementary Materials

The following supporting information can be downloaded at: https://www.mdpi.com/article/10.3390/nano14221822/s1, Figure S1. (a) Multipolar decomposition of a silicon nanocylinder of *R* = 110 nm and *H* = 50 nm. (b) Multipolar decomposition of a MRI nanocylinder of *R* = 220 nm and *H* = 200 nm. Vertical grey lines correspond to the emission and excitation wavelengths. Figure S2. Scattering efficiencies for (a) a dimer of silicon nanocylinders of *R* = 110 nm and *H* = 50 nm, and (b) a dimer of MRI nanocylinders of *R* = 220 nm and *H* = 200 nm. Vertical grey lines correspond to the emission and excitation wavelengths. Table S1. Results on non-hybrid and hybrid dimers combining the non-optimal sizes for the HRI and MRI dielectric NPs of *R* = 110 nm, *H* = 50 nm and *R* = 220 nm, *H* = 200 nm respectively. Figure S3. Scheme of all QDs positions considered with respect to the silicon-gold dimer. The size for the silicon NP is *R* = 80 nm and *H* = 120 nm and for the gold NP *R* = 50 nm and *H* = 150 nm. Figure S4. Near-field map corresponding to the XY-plane, 5 nm above the substrate, at the (a) excitation (570 nm) and (b) emission (650 nm) wavelengths, for the silicon-gold dimer showing the highest electric field enhancement between the nanocylinders. Table S2. Results on Purcell factor, radiation efficiency, electric field enhancement and photoluminescence signal enhancement for the different QDs positioned with respect to the gold-silicon dimer. Figure S5. Multipolar decomposition for a MRI NP slightly smaller than the optimal size (*R* = 140 nm and *H* = 200 nm). Table S3. Comparison of the results between optimal dimers and dimers involving a slightly smaller MRI NP. Figure S6. Purcell factor and electric field confinement dependence on the gap distance analyzed for the optimal MRI-gold dimer (*R* = 150 nm and *H* = 200 nm for the MRI NP and *R* = 50 and *H* = 150 nm for the gold NP). Figure S7. Near-field maps in the XZ-plane cutting at the center of the dimers, at the excitation (570 nm) wavelength for the (a) MRI-MRI and (b) Si-gold truncated cone dimers compared to (c) MRI-MRI and (d) Si-gold cylindric dimers. The sizes considered were the optimal ones described in the manuscript: *R* = 150 nm and *H* = 200 nm for the MRI NP, *R* = 50 nm and *H* = 150 nm for the gold NP and *R* = 120 nm and *H* = 80 nm for the silicon NP. Table S4. Comparison of results between perfect aligned nanocylinders and imperfect truncated nanocones. Table S5. Comparison between the results obtained in this study and the presented at [38]. Refs [38,39,45,46] are cited in the supplementary materials.

## Abbreviations

The following abbreviations are used in this manuscript:

FEM	Finite element method
HRI	High refractive index
LDOS	Local density of states
MRI	Moderate refractive index
NP	Nanoparticle
PML	Perfectly matched layer
QD	Quantum dot
