# Supplementary material for: An Evaluation of Moderate-Refractive-Index Nanoantennas for Enhancing the Photoluminescence Signal of Quantum Dots
Source: Nanomaterials (Basel). 2024 Nov 14;14(22):1822. doi: 10.3390/nano14221822 (PMC11597763; doi:10.3390/nano14221822)
Supplement: Supplementary file 1 [file nanomaterials-14-01822-s001.zip › nanomaterials-3267744-supplementary.pdf]

Supplementary information

An Evaluation of Moderate-Refractive-Index Nanoantennas for Enhancing the Photoluminescence Signal of Quantum Dots

Rafael Ramos Uña, Braulio García Cámara and Ángela I. Barreda **

Affiliation: Department of Electronic Engineering, University Carlos III of Madrid, Avda. de la Universidad, 30, 28911 Leganés, Spain

*Corresponding author: raramosu@ing.uc3m.es (R.R.U.); abarreda@ing.uc3m.es (Á.I.B.)

SI-1. Multipolar decomposition

The multipolar decomposition of the light scattering of a nanoparticle can be derived from the current density distribution obtained from a finite-element-method simulation (COMSOL Multiphysics). The following process is described in [1].

$$p_i = \frac{i}{\omega}\int \mathbf{J}_i \, dv \quad \text{Eq. S1}$$

$$m_j = \frac{1}{2}\int (\mathbf{r} \, x \, \mathbf{J})_j \, dv \quad \text{Eq. S2}$$

$$\bar{\bar{Q}}_{jk}^{(e)} = \frac{i}{\omega}\int r_j \, \mathbf{J}_k + r_k \, \mathbf{J}_i - \frac{2}{3}\delta_{jk}(\mathbf{r} \cdot \, \mathbf{J}) \, dv \quad \text{Eq. S3}$$

$$\bar{\bar{Q}}_{jp}^{(m)} = \frac{2}{3}\int (r_p(\mathbf{r} \, x \, \mathbf{J})_j + r_j(\mathbf{r} \, x \, \mathbf{J})_p) \, dv \quad \text{Eq. S4}$$

Where *p* and *m* are the electric and magnetic dipolar contributions respectively, and Q^e and Q^m the quadrupolar electric and magnetic contributions. Equations 1 to 4 correspond to the primitive Cartesian multipoles, being **J** the current density in an infinitesimal volume, **r** = {r_1, r_2, r_3} the vector from the origin of coordinates to an arbitrary point of the current distribution area and δ_{12} is a Kronecker delta symbol.

Then, the scattered power can be calculated as:

$$\begin{aligned} P_{\text{scat}} = &\frac{k^4\sqrt{\varepsilon_\text{d}}}{12\pi \cdot \varepsilon_0^2 \cdot \text{c} \cdot \mu_0}\left|p_i + \frac{\text{i} \cdot k \cdot \varepsilon_\text{d}}{\text{c}} T_i^{(e)}\right|^2 \\ &+ \frac{\text{k}^4 \cdot \varepsilon_\text{d}\sqrt{\varepsilon_\text{d}}}{12\pi \cdot \varepsilon_0 \cdot \text{c}}\left|m_i + \frac{\text{i} \cdot k \cdot \varepsilon_\text{d}}{\text{c}} T_i^{(m)}\right|^2 \\ &+ \frac{k^6 \cdot \varepsilon_\text{d}\sqrt{\varepsilon_\text{d}}}{160\pi \cdot \varepsilon_0^2 \cdot \text{c} \cdot \mu_0}\left|Q_{ij}^{(e)}\right| + \frac{k^6 \cdot \varepsilon_d^2\sqrt{\varepsilon_\text{d}}}{160\pi \cdot \varepsilon_0 \cdot \text{c}}\left|Q_{ij}^{(m)}\right| \end{aligned} \quad \text{Eq. S5}$$

Where *k* is the wavevector, μ_0, ε_0 and c are the magnetic permeability, the electric permittivity and the speed of light in vacuum, ε_d is the magnetic permeability of the surrounding material and $T_i^{(e)}$ and $T_i^{(m)}$ are the toroidal contributions to the electric dipole and magnetic dipoles respectively, given by the following equations:

$$T_j^{(e)} = \frac{1}{10}\int (\mathbf{J} \cdot \mathbf{r})r_j - 2r^2\mathbf{J}_j \, dv \quad \text{Eq. S6}$$

$$T_j^{(m)} = \frac{i\omega}{20}\int r^2(\mathbf{r} \text{ x } \mathbf{J})_j \, dv \quad \text{Eq. S7}$$

The scattering cross-section (σ_{scat}) is then calculated as:

$$\sigma_{\text{scat}} = 2\sqrt{\frac{\mu_0}{\varepsilon_0\varepsilon_\text{d}}\frac{P_{\text{scat}}}{|\boldsymbol{E}_{\text{inc}}|^2}} \quad \text{Eq. S8}$$

Where $|E_{inc}|^2$ is the squared modulus of the incident field. And finally, the scattering efficiency is given by the normalization of the scattering cross-section on the geometric cross-section of the scatterer:

$$Q_{scat} = \frac{\sigma_{scat}}{\sigma_{geom}} \qquad \text{Eq. S9}$$

In the case of a single cylinder σ_{geom} is equal to $\pi \cdot r^2$, where r is the NP radius.

SI-2. Considered alternative sizes for the nanocylinders

The geometrical range of the nanocylinders has been restricted, firstly due to fabrication limitations, and secondly to maintain them in a subwavelength regime. Taking this into account, we have considered a height range between 20 nm and 200 nm, and a radius range from 50 nm to 300 nm, with steps of 30 nm and 50 nm respectively. Considering all the performed simulations, the sizes for the nanoparticles were chosen with the objective of obtaining Mie resonances in the wavelength range of interest (570-650 nm) Some other sizes also demonstrated Mie resonances in that range but, due to worse results for the photoluminescence enhancement, they were discarded as final results. For all cases the NPs were illuminated by a plane wave linearly polarized along the *x*-axis and propagating in the negative direction of the z-axis towards the nanostructure. The multipolar decomposition for two of the discarded sizes are shown at Supplementary Figure S1.

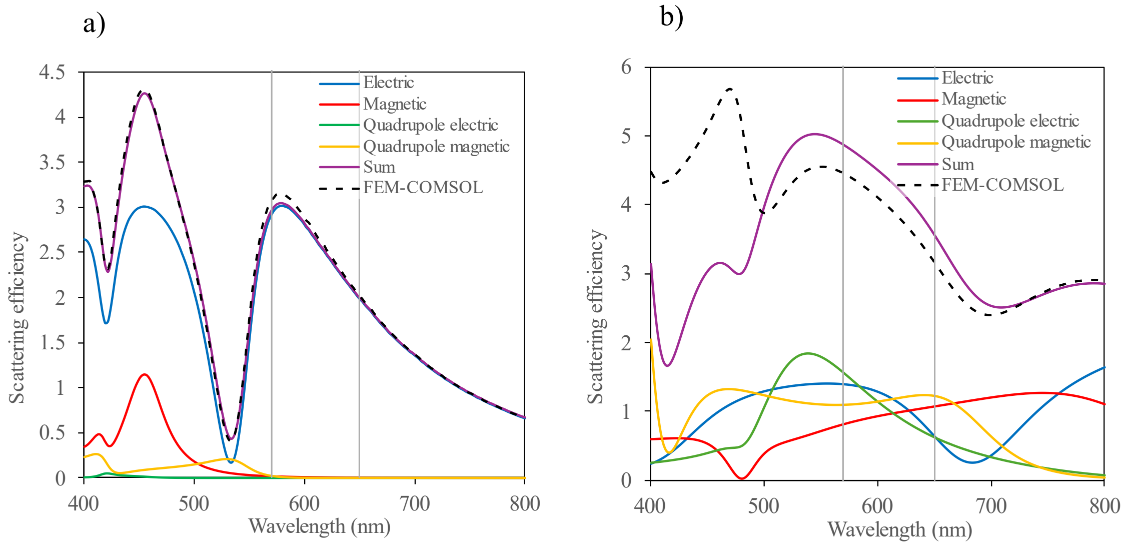

Supplementary Figure S1. a) Multipolar decomposition of a silicon nanocylinder of *R*=110nm and *H*=50nm. b) Multipolar decomposition of a MRI nanocylinder of *R*=220nm and *H*=200nm. Vertical grey lines correspond to the emission and excitation wavelengths.

At first sight, the multipolar decomposition from the silicon nanocylinder in Supplementary figure S1a) suggests that even though there is a Mie resonance almost covering the desired wavelength range, the scattering efficiency may not be high enough compared to that of the selected size (see Fig. 3b). Additionally, there are not resonances at both the excitation and emission wavelengths of the quantum dot (QD), observing just one dipolar electric resonance close to the excitation wavelength. All in all, this leads to a decrease in the PL

signal enhancement with respect to the optimal case, where a dipolar electric/magnetic resonance is excited at the excitation/emission wavelengths of the quantum emitter.

In contrast to the silicon NP, the multipolar decomposition from the MRI nanocylinder, Supplementary figure S1b), demonstrates the opposite behavior. It shows Mie resonances in the scattering efficiency for the desired wavelength range, with not only an overlap of dipole electric and dipole magnetic resonances but also the appearance of electric and magnetic quadrupoles, indicating that the optimal size for the nanocylinder must be smaller to avoid the excitation of high multipolar orders.

Following the steps of the manuscript, the results for the scattering efficiency of the homogeneous dimers for the non-optimal sizes of HRI and MRI are shown in Supplementary Figure S2. The dimers were illuminated by a plane wave linearly polarized in the *x*-axis and propagating in the negative direction of the *z*-axis towards to the NPs.

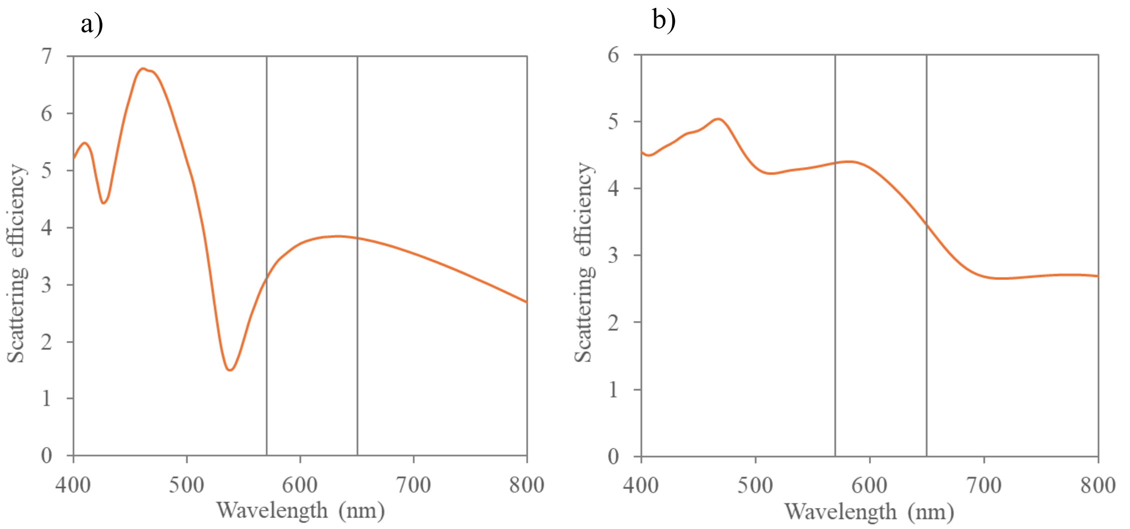

Supplementary Figure S2. Scattering efficiencies for a)a dimer of silicon nanocylinders of *R*=110 nm and *H*=50 nm, and b)a dimer of MRI nanocylinders of *R*=220 nm and *H*=200 nm. Vertical grey lines correspond to the emission and excitation wavelengths.

The scattering efficiency shown for both dielectric dimers in Supplementary Figure S2 demonstrates wide Mie resonances at the excitation and emission wavelengths (labelled with vertical lines in the figure).However, they may not be as high as those for the most optimal non-hybrid dimers of MRI and HRI described in the manuscript.

The values for the excitation and emission enhancement have also been calculated for the non-hybrid dimers of non-optimal HRI and MRI NPs. In addition, this analysis has also been carried out for hybrid dimers combining the non-optimal HRI/MRI NP with the optimal Au/MRI/HRI NP (see Supplementary Table S1).

Supplementary Table S1. Results on non-hybrid and hybrid dimers combining the non-optimal sizes for the HRI and MRI dielectric NPs of R=110 nm, H=50 nm and R=220 nm, H=200 nm respectively.

		Materials	NP 1		NP 2		F	η	$\lvert E\rvert/\lvert E_0\rvert$	$\lvert E\rvert/\lvert E_0\rvert^2 \cdot F$
			R_1 [nm]	H_1 [nm]	R_2[nm]	H_2 [nm]				
Non-hybrid dimers	#1	MRI-MRI	220	200	220	200	7.02	1	1.82	23
	#2	Si-Si	110	50	110	50	25.58	0.96	3.92	392
Hybrid dimers	#3	MRI-Au	220	200	50	150	95.12	0.63	4.51	1935
	#4	Si-Au	110	50	50	150	115.30	0.69	6.14	4351
	#5	MRI-Si	220	200	80	120	15.90	0.96	3.72	220
	#6	MRI-Si	220	200	110	50	14.55	0.97	2.69	105
	#7	MRI-Si	150	200	110	50	14.32	0.97	3.54	179

As expected, the results for the dimers combining these non-optimal sizes for the nanocylinders do not show better performance than those described in the main manuscript, therefore demonstrating worse performance and justifying why these are considered non-optimal.

SI-3. Quantum dot positioning

While the initial position for the QD was in the middle of the gap between the NPs and on top of the substrate, a study on excitation and emission enhancement based on the QD position has been conducted. Supplementary Figure S3 shows the different QD positions considered studied for the optimal silicon-gold dimer described in the manuscript. The dimer was illuminated by a plane wave linearly polarized along the x-axis and propagating in the negative direction of the z-axis. In Supplementary Figure S4, the electrical near-field distribution, which explains the difference in the electric field enhancement for each position, are shown. Supplementary Table S2 summarizes the electric field enhancement, Purcell factor, radiation efficiency, and photoluminescence signal enhancement for the different QD positions in the gold-silicon dimer. These results clearly show that the position considered is the optimum one.

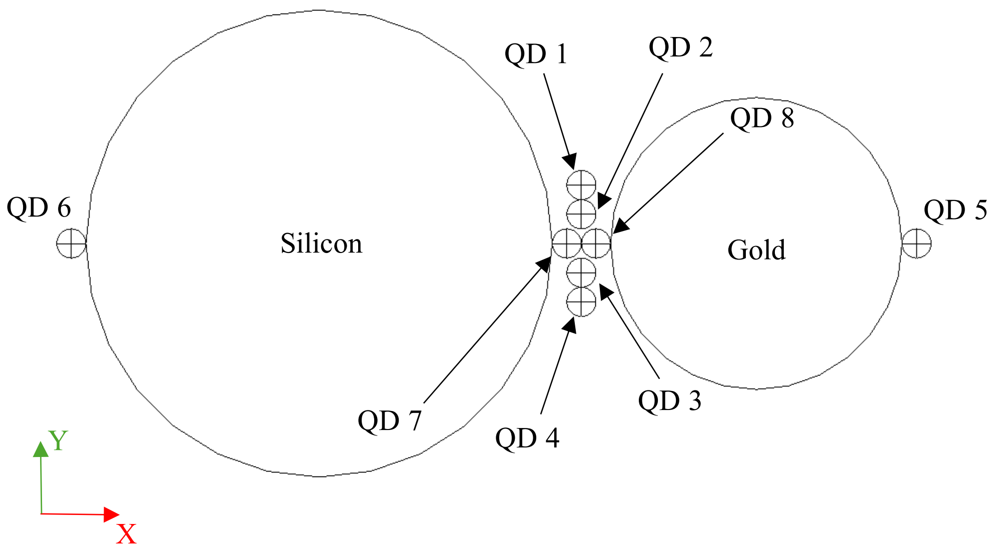

Supplementary Figure S3. Scheme of all QDs positions considered with respect to the silicon-gold dimer. The size for the silicon NP is *R*=80 nm and *H*=120 nm and for the gold NP *R*=50 nm and *H*=150 nm.

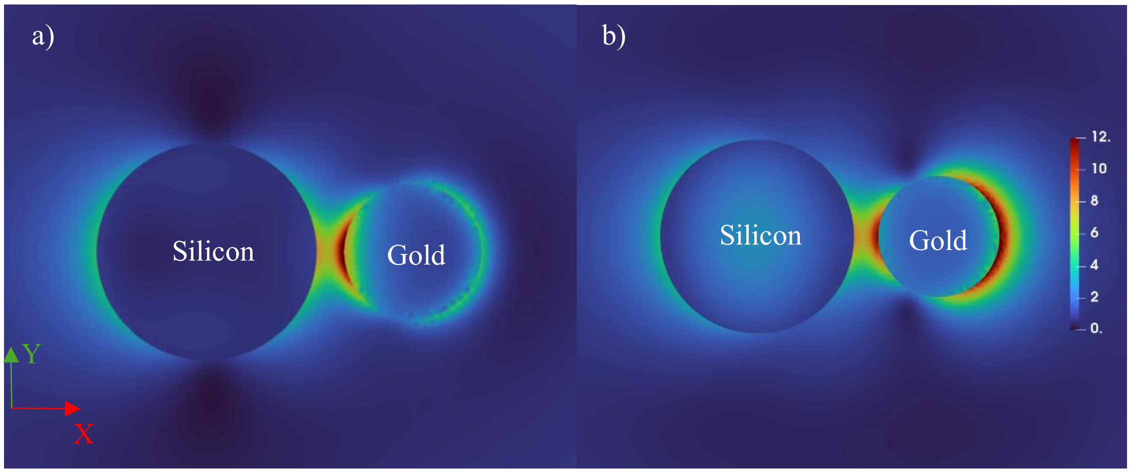

Supplementary Figure S4. Near-field map corresponding to the XY-plane, 5 nm above the substrate, at the a) excitation (570 nm) and b) emission (650 nm) wavelengths, for the silicon-gold dimer showing the highest electric field enhancement between the nanocylinders.

Supplementary Table S2. Results on Purcell factor, radiation efficiency, electric field enhancement and photoluminescence signal enhancement for the different QDs positioned with respect to the gold-silicon dimer.

QD	F	η	$\|E\|/\|E_0\|$	$\|E\|/\|E_0\|^2 \cdot F$
Center	138.39	0.72	7.44	7660
#1	65.18	0.73	5.08	1683
#2	108.35	0.72	6.70	4870
#3	108.38	0.72	6.71	4889
#4	65.26	0.73	5.04	1660
#5	164.08	0.37	3.17	1653
#6	108.38	0.77	3.93	205
#7	116.72	0.77	7.21	6068
#8	261.64	0.53	9.645	24339

The highest electric field enhancement is achieved for the QDs positioned in the middle of the gap (#1-4 and #7-8). Specifically, the one closer to the gold cylinder (#8). However, as expected, the radiation efficiency decreases as the QD gets closer to the gold NP, due to the increase in the non-radiative power. On the other hand, the QD more proximate to the silicon NP (#7) experiences less losses, but lower electric field enhancement. Also, the QDs displaced in the *y*-axis from the center (#5-6) experience a large decrease in the photoluminescence. Therefore, the optimal position for the quantum dot (QD) may be at the center of the gap, as it represents a balanced compromise between achieving high enhancement and maximizing radiation efficiency.

SI-4. Fabrication challenges

During the fabrication process, some defects may appear in the nanostructures. In this section, their influence on the results is individually explained.

SI-4.1. Small size changes

Small changes in the fabrication of the nanocylinders may not significantly influence the results, as the proposed systems exhibit broadband excited resonances. This means that minor variations in the dimensions of the NPs, even if they cause a spectral shift of the resonant wavelength, will not lead to substantial changes in the values of emission and excitation enhancement. To demonstrate this statement, simulations over changes in the MRI NP have been performed. The results on the multipolar decomposition can be found in Supplementary Figure S5, while a comparison on the Purcell factor, radiation efficiency, electric field enhancement and photoluminescence signal enhancement between dimers involving the optimized size of the NPs, and dimers simulating a slightly smaller MRI NP are represented in the Supplementary Table S3.

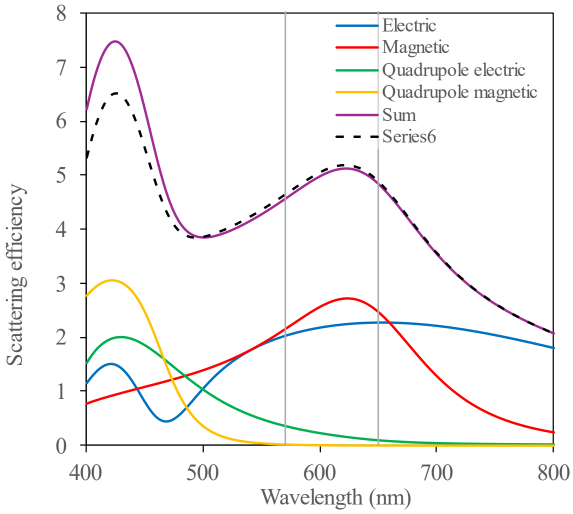

Supplementary Figure S5. Multipolar decomposition for a MRI NP slightly smaller than the optimal size (R = 140 nm and H = 200 nm).

Supplementary Table S3. Comparison of the results between optimal dimers and dimers involving a slightly smaller MRI NP.

		Materials	F	η	$\|E\|/\|E_0\|$	$\|E\|/\|E_0\|^2 \cdot F$
Perfect cylinders' size	#1	MRI-MRI	7.63	1	3.18	77
	#2	MRI-Si	18.31	0.97	4.06	302
	#3	MRI-Au	109.47	0.66	4.64	2361
Slightly smaller MRI	#4	MRI-MRI	7.52	1	3.11	73
	#5	MRI-Si	17.42	0.97	3.82	254
	#6	MRI-Au	101.44	0.65	4.51	2062

SI-4.2. Gap defect

As explained in the main manuscript, the distance between the nanocylinders is maintained at over 20 nm due to fabrication limits. The increase in the gap distance impacts negatively on the results concerning excitation and emission enhancement, as demonstrated in Supplementary Figure S6, so a trade-off between fabrication limitations and enhancement, due to the interaction between the NPs, justifies the smallest feasible gap. A defect in the gap distance may cause a decrease in photoluminescence signal enhancement. Additionally, the displacement of the QD due to a defect in the gap may also lead to negative outcomes, as explained in SI-3.

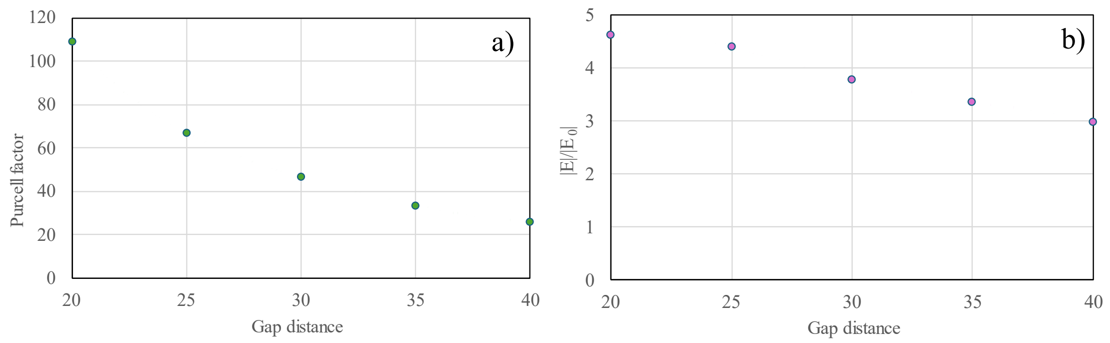

Supplementary Figure S6. Purcell factor and electric field confinement dependence on the gap distance analyzed for the optimal MRI-gold dimer (*R*=150 nm and *H*=200 nm for the MRI NP and *R*=50 and *H*=150 nm for the gold NP).

SI-4.3. Truncated cone shaped cylinders

The final defect considered was the potentially non-straight walls of the cylinders, resulting in truncated cone-shaped NPs. A tilt of 2.2º with respect to the normal was considered, as this inclination is sufficient to perceive the walls as inclined. The studied dimers correspond to the same sizes as the optimal ones for the MRI-MRI (*R*=150 nm and *H*=200 nm) and the silicon-gold dimers described in the manuscript (*R*=80 nm and *H*=120 nm for the silicon and *R*=50 nm and *H*=150 nm for the gold NP). The truncated cone dimers were studied under the same conditions as the cylindrical dimers, illuminated by a plane wave linearly polarized along the axis that joints both NPs, and propagating in the negative direction of the *z*-axis. The results related to this defect are presented in Supplementary Table S4. It can be observed a decrease in photoluminescence signal enhancement in comparison to perfectly straight cylinders. Near-field maps for the considered dimers are represented in Supplementary Figure S7.

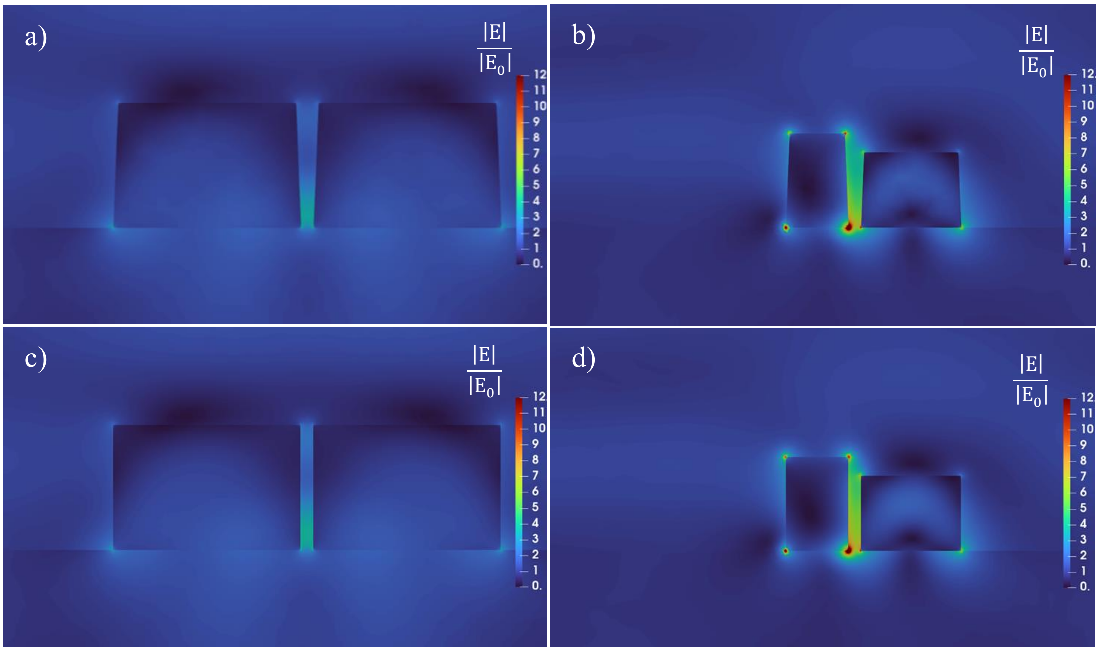

Supplementary Figure S7. Near-field maps in the XZ-plane cutting at the center of the dimers, at the excitation (570 nm)wavelength for the a)MRI-MRI and b)Si-gold truncated cone dimers compared to c)MRI-MRI and d)Si-gold cylindric dimers. The sizes considered were the optimal ones described in the manuscript: *R* = 150 nm and *H* = 200 nm for the MRI NP, *R* = 50 nm and *H* = 150 nm for the gold NP and *R* = 120 nm and *H* = 80 nm for the silicon NP.

Supplementary Table S4. Comparison of results between perfect aligned nanocylinders and imperfect truncated nanocones.

		Materials	F	η	$\lvert E\rvert/\lvert E_0\rvert$	$\lvert E\rvert/\lvert E_0\rvert^2 \cdot F$
Cylinders	#1	MRI-MRI	7.63	1	3.18	77
	#2	Si-Au	138.39	0.72	7.44	7660
Truncated cones	#3	MRI-MRI	6.63	1	3.04	61
	#4	Si-Au	134.41	0.7	7.17	6919

SI-5. Comparison to previous results

To provide a clear reference for the results obtained, it is useful to contrast them with previous findings on the enhancement of QD photoluminescence. For this purpose, the results presented in references [2-4] will be utilized, as these works study the excitation and emission enhancement of QDs with similar excitation and emission wavelengths as the considered in this work. The comparison is shown at Supplementary Table S5.

The referenced structures, correspond to a silicon nanoring and a silicon nanosphere separated from a gold mirror by a thin alumina (Al_2O_3)layer (10 nm)in the case of the references [2] and [3] respectively, and a hybrid nanoantenna consisting of and inner metal nanodisk and an outer dielectric ring cavity in the case of the reference [4]. These results

show some variation in values due to the difference in geometry. However, they are still comparable and have values of the same order of magnitude with respect to those obtained in this work.

Supplementary Table S5. Comparison between the results obtained in this study and the presented at [2].

		Materials	Gap [nm]	*F*	*η*	|*E*|/|*E*0|
Dimers of this work	#1	Au-Au	20	285.64	0.66	8.612
	#2	MRI-MRI	20	7.63	1	3.18
	#3	Si-Si	20	31.8	0.96	5.39
	#4	Au-Si	20	138.39	0.72	7.44
Results from [2]	#5	Si over Au	10	206.2	0.29	7.3
Results from [3]	#6	Si over Au	10	>120	>0.8	>6
Results from [4]	#7	Si-Au-Si	10	80	0.4	7.78

References

[1] Gurvitz, E.A.; Ladutenko, K.S.; Dergachev, P.A.; Evlyukhin, A.B.; Miroshnichenko, A.E.; Shalin, A.S. The High-Order Toroidal 362 Moments and Anapole States in All-Dielectric Photonics. Laser Photonics Rev. 2019, 13, 1800266. https://doi.org/https:363//doi.org/10.1002/lpor.201800266. 364

[2] Dmitriev, P.A.; Lassalle, E.; Ding, L.; Pan, Z.; Neo, D.C.J.; Valuckas, V.; Paniagua-Dominguez, R.; Yang, J.K.W.; Demir, H.V.; Kuznetsov, A.I. Hybrid Dielectric-Plasmonic Nanoantenna with Multiresonances for Subwavelength Photon Sources. ACS Photonics 2023, 10, 582–594. https://doi.org/10.1021/acsphotonics.2c01332.

[3] Yang, G.; Niu, Y.; Wei, H.; Bai, B.; Sun, H.B. Greatly amplified spontaneous emission of colloidal quantum dots mediated by a dielectric-plasmonic hybrid nanoantenna. Nanophotonics, 8, 12, 2019, 2313-2319. https://doi.org/10.1515/nanoph-2019-0332

[4] Sun, S.; Zhang, T.; Liu, Q.; Ma, L.; Du, Q.; Duan, H. Enhanced Directional Fluorescence Emission of Randomly Oriented Emitters via a Metal–Dielectric Hybrid Nanoantenna. J. Phys. Chem. C. 2019, 123 (34), 21150-21160 https://doi.org/10.1021/acs.jpcc.9b06280
